# Exploring the efficacy of using hypertonic saline for nebulizing treatment in children with bronchiolitis: a meta-analysis of randomized controlled trials

**DOI:** 10.1186/s12887-020-02314-3

**Published:** 2020-09-14

**Authors:** Chia-Wen Hsieh, Chiehfeng Chen, Hui-Chuan Su, Kee-Hsin Chen

**Affiliations:** 1 Department of Nursing, Wan Fang Hospital, Taipei Medical University, Taipei, Taiwan; 2 Center for Nursing and Healthcare Research in Clinical Practice Application, Wan Fang Hospital, Taipei Medical University, Taipei, Taiwan; 3 Department of Public Health, School of Medicine, College of Medicine, Taipei Medical University, Taipei, Taiwan; 4 Cochrane Taiwan, Taipei Medical University, Taipei, Taiwan; 5 Division of Plastic Surgery, Department of Surgery, Wan Fang Hospital, Taipei Medical University, No.111, Sec. 3, Xinglong Rd., Wenshan Dist., Taipei City, 116 Taiwan, Republic of China; 6 Evidence-based Medicine Center, Wan Fang Hospital, Taipei Medical University, Taipei, Taiwan; 7 Post-Baccalaureate Program in Nursing, College of Nursing, Taipei Medical University, Taipei, Taiwan

**Keywords:** Bronchiolitis, Children, Hypertonic saline, Nebulizer treatment, Length of hospital stay, Efficacy

## Abstract

**Background:**

Inhaled hypertonic saline (HS) has shown benefit in decreasing airway edema in acute bronchiolitis which is the most common lower respiratory infection resulting in dyspnea among infants under 2 years old. The aim of this systematic review and meta-analysis was to evaluate the efficacy and safety of HS in the implementation of treatment with nebulized HS among children with bronchiolitis.

**Methods:**

A systematic literature search was conducted using Cochrane Library, PubMed, EMBASE and Airiti Library (Chinese Database) for randomized controlled trials from inception to July 2019. We calculated pooled risk ratios (RR), mean difference (MD) and 95% CI using RevMan 5.3 for meta-analysis.

**Results:**

There were 4186 children from 32 publications included. Compared to the control group, the HS group exhibited significant reduction of severity of respiratory distress, included studies used the Clinical Severity Score (*n* = 8; MD, − 0.71; 95% CI, − 1.15 to − 0.27; *I*^2^ = 73%) and full stop after Respiratory Distress Assessment Instrument (*n* = 5; MD, − 0.60; 95% CI, − 0.95 to − 0.26; *I*^2^ = 0%) for evaluation respectively. Further, the HS group decreased the length of hospital stay 0.54 days (*n* = 20; MD, − 0.54; 95% CI, − 0.86 to − 0.23; *I*^2^ = 81%).

**Conclusions:**

We conclude that nebulization with 3% saline solution is effective in decreasing the length of hospital stay and the severity of symptoms as compared with 0.9% saline solution among children with acute bronchiolitis. Further rigorous randomized controlled trials with large sample size are needed.

## Background

Bronchiolitis is the most common lower-respiratory infection in infants, affecting 68.8% of infants and neonates aged < 12 months [1, 2], and is a major cause of hospitalization in children during the first year of life [3, 4]. Bronchiolitis is primarily caused by viral infection which results in inflammation of the bronchioles in the lungs [5, 6]. The infection can last 2 ~ 3 weeks, and causes mucosal congestion and sputum secretion during the disease course [7, 8]. Common symptoms include excessive coughing with tachypnea, fever and wheezing [1, 9].

In case of severe nasal congestion, a child might resort to open-mouth breathing and prone to dyspnea caused by tracheal obstruction, which may cause respiratory failure in severe cases [10, 11]. Infants may be prone to vomit due to frequent coughing at night that affect their sleeping quality, day time activities and mental status as well as recovery of the body’s immune system [12–15].

Approximately 50–80% of bronchiolitis are caused by respiratory syncytial virus (RSV), thereby, treatment by antibiotics is usually ineffective [16]. According to the 2014 American Academy of Pediatrics Bronchiolitis guideline, the primary treatment method is supportive, such as rest, maintain nutrition intake and fluid supplementation [17, 18]. For symptoms such as cough and fever, the use of supportive medications such as antitussive syrup, antipyretics or nebulizer can help relieve the symptoms [18–20].

With the use of normal saline as the diluent in nebulizers and the oxygen as vaporizer, the water molecules or drugs can be breathed through the mouth or nose and spread to the respiratory tract and lungs by the airflow. After the alveolar capillaries absorb the molecules, the drugs can dilute the secretions in the respiratory tract, then induce expectoration and relieve symptoms of bronchospasm [21, 22].

Recently, several studies pointed out that hypertonic saline (3%) is beneficial in inducing the penetration of water molecules into the lung mucosa, allowing the bronchial mucosa or submucosal layers to absorb water molecules and reduce the possibility of edema of the airway [23, 24]. It also uses the principle of vaporization to moisturize the airway surface, increase mucosa cilia function, and accelerate elimination of obstructive sputum to achieve better treatment effects [25]. However, other studies also pointed out that there is no significant difference in efficacy between hypertonic saline and normal saline nebulizers for treating children with bronchiolitis [26–28]. A systematic literature review and meta-analysis by Zhang et al. [29] demonstrated that the use of hypertonic saline can significantly shorten the length of hospital stay, but the article did not provide an explanation for the high heterogeneous results.

The purpose of this study is to conduct a systematic review and meta-analysis of the latest randomized controlled trials (RCTs) to update the effectiveness and safety of using hypertonic saline (3%) for nebulizing treatment in children with bronchiolitis, and we included results of a children’s sleep index in the analysis, with the aim to provide a reference for clinical treatment.

## Methods

### Database searches

We found Mesh terms and related synonyms through the PubMed Mesh Database and used Boolean logic to search for literatures. Keywords and searching strategy were as follows: “bronchiolitis” OR “pediatrics” OR “child*” AND “3% saline” OR “hypertonic saline” AND “saline solution” OR “0.9% saline” OR “normal solution.” The study screened the following online databases: Cochrane, PubMed, EMBASE, and Airiti Library. The search period was any publications before July 2019. Only publications in English and Chinese were included. Additionally, we manually searched the literatures cited in related systematic literature reviews and RCTs.

### Inclusion criteria

Two independent researchers (CW Hsieh and HC Su) screened the literatures. The inclusion criteria were as follows: (1) population: children aged < 18 years with bronchiolitis; (2) intervention: hypertonic saline (3%); (3) control intervention: normal saline (0.9%); (4) results: severity of respiratory distress, length of hospital stay (LOS), rate of hospitalization, rate of readmission, time of sleeping, frequency of waking up in the night, drug side effects, etc.; and (5) study design: RCTs. Exclusion criteria were patients with other comorbidities such as congenital respiratory tract disease, cardiac insufficiency and immunodeficiency. During the screening process of browsing through the titles, abstracts, and full articles, any different opinions that emerged, a third researcher (KH Chen or C Chen) joined the discussion, and a decision was made through consensus opinion.

### Literature quality assessment

Two researchers (CW Hsieh and HC Su) used the Cochrane risk of bias tool (RoB) 2.0 to independently conduct a literature risk assessment. The five fields for assessment included (1) Bias arising from the randomization process; (2) bias due to deviations from intended interventions; (3) bias due to missing outcome data; (4) bias in measurement of the outcome; and (5) bias in selection of the reported result. The assessment results were rated as low, some concern, and high risk of bias. According to suggestions by the Cochrane handbook for systematic reviews of interventions, if any one of the fields in the result indices were assessed as having high risk of bias, then the overall assessment of the study would be labeled as high risk.

Next, the Grading of Recommendations Assessment, Development and Evaluation (GRADE) system was used for assessing the evidence body of the included meta-analytical results. Trials included by this study were randomized controlled trials; therefore, the preliminary assessment for evidence level was high, and the assessment was graded based on five downgrade factors, which included risk of bias, inconsistency, indirectness, imprecision, and publication bias. The final quality of evidence was graded as either a high, moderate, low, or very low level. Finally, clinical recommendations were formed according to factors such as the strength of the evidence, clearness of intervention pros and cons, patient preference, and resources, and the recommendation strength was graded as either strong or weak.

### Data analysis

Two researchers (CW Hsieh and HC Su) independently extracted research data and conducted a meta-analysis using the Revman 5.3 software (The Nordic Cochrane Centre, Copenhagen, Denmark, 2014). Mean and standard deviation (SD) values were extracted for continuous data, and number of people in each group and number of incidences were extracted to analyze categorical data. The Cochrane Q and *I*^2^ tests were used to assess heterogeneity. When the Q value showed significant difference (*p* < 0.1), it was considered heterogeneity existed in the study samples. The *I*^2^ test was used to determine the level of heterogeneity between the study samples, and the final results were collectively portrayed in a forest plot to exhibit the effect size and 95% confidence interval (CI).

### Sensitivity analysis

The meta-analysis results were cautiously assessed, and if high heterogeneity was noted among the results, then sensitivity and subgroup analyses were conducted. Subgroups were divided based on factors such as the study’s research region, hospitalization, and LOS, and the obtained results were compared with results before subgrouping to confirm the stability of the meta-analytical results.

## Results

### Literature search results

**Fig. 1:**
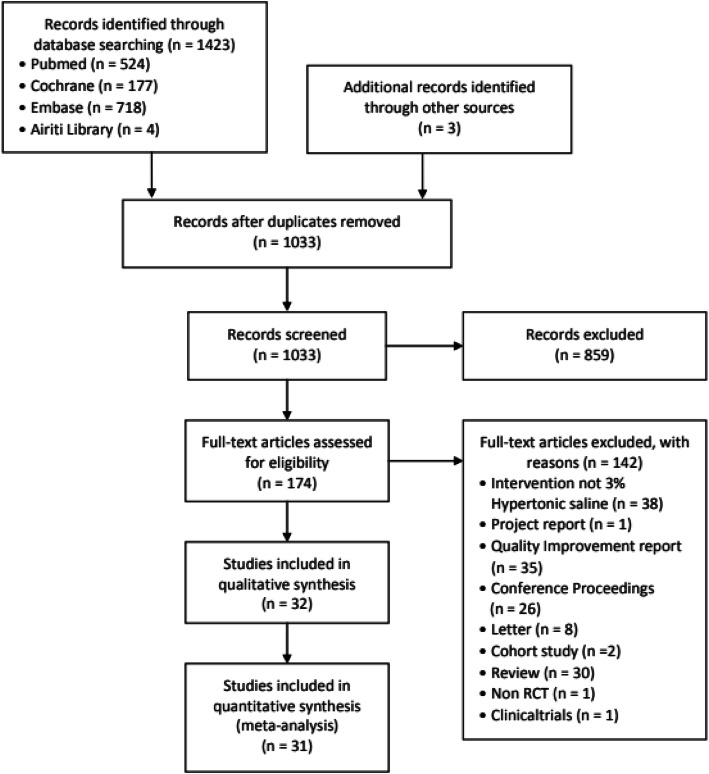
Flow diagram displaying the search process and search results

In total, 1423 articles were found in the databases, and 3 articles were manually searched; 1033 articles remained after 393 duplicate articles were excluded; 859 articles were excluded after the titles and abstract were incompatible with the study; and 174 articles were included for careful examination of the full texts. Finally, 32 RCTs [8, 27, 28, 30–58] along with 31 studies were included in the meta-analysis. Details of the search and screening process of articles and reasons for exclusion are presented in Fig. 1.

### Characteristics of included studies

Of the 32 selected RCTs, 20 (62.5%) were conducted in the Asian region, and six (18.8%) were conducted in the Americas or European countries. Regarding the research setting, 22 (68.8%) studies were conducted in hospital wards with study targets being hospitalized children, and 10 studies (31.3%) were conducted in emergency wards of outpatient departments.

All 4186 included subjects were diagnosed with acute bronchiolitis, 70.5% of subjects had RSV infection, two had a past history of asthma, 2100 (50.2%) were treated with hypertonic saline (3%), and 2086 (49.8%) were treated with normal saline. The mean age of the two population groups were 6.3 months vs. 6.5 months, the sex ratio were 58.3% males vs. 41.7% females, and there were no significant differences regarding the age or sex between these two groups (*p* > 0.05). Dosages of saline used for nebulizing treatment differed according to each study’s design, and the dosage used ranged 2 ~ 5 ml. Regarding required treatments according to different clinical symptoms, 22 studies (68.8%) combined treatment with epinephrine, bronchodilators, or steroids. The basic characteristics of the included studies are summarized in Table 1.

**Table 1:** Characteristics of the included trials

Study	Patients		Intervention		Comparison		Outcome
year		Average age (male %)	3% H/S	Additional drugs	0.9% N/S	Additional drugs	
Country		RSV positive rate					
Al-Ansari et al. 2010 [30] [?]Saudi Arabia	< 18 mon Inpatients *n* = 114	3.9 mon (59.1%) No information	5 mL (*n* = 58)	+ 1.5 mg epinephrine	5 mL (*n* = 56)	+ 1.5 mg epinephrine	LOS CSS
Angoulvant et al. 2017 [31] France	6 wk. ~ 12 mon ED *n* = 772	3 mon (60.2%) 86.4%	4 mL (*n* = 385)		4 mL (*n* = 387)		RDAI ROH Adverse events
Anil et al. 2010 [32] Turkey	6 wk. ~ 24 mon ED *n* = 149	9.5 mon (64.5%) No information	1) 4 mL (*n* = 39) 2) 4 mL (*n* = 36)	+ 1.5 mg epinephrine + 2.5 mg salbutamol	1) 4 mL (*n* = 38) 2) 4 mL (*n* = 36)	+ 1.5 mg epinephrine + 2.5 mg salbutamol	ROH ROR
Everard et al. 2014 [8] UK	< 12 mon Inpatients *n* = 291	3.4 mon (54.5%) 61.5%	4 mL (*n* = 142)	+ standard care	(*n* = 149) *Nebulizer use not reported	+ standard care	LOS RDAI
Flores et al. 2016 [27] Portugal	< 12 mon Inpatients *n* = 68	3.6 mon (52.9%) 85.4%	3 mL (*n* = 33)	+ 1.25 mg salbutamol	3 mL (*n* = 35)	+ 1.25 mg salbutamol	LOS CSS
Florin et al. 2014 [28] USA	2 ~ 24 mon ED *n* = 62	6.7 mon (45.2%) No information	4 mL (*n* = 31)		4 mL (*n* = 31)		ROH
Grewal et al. 2009 [33] Canada	6 wk. ~ 12 mon ED *n* = 46	5 mon (60.9%) 82.2%	2.5 mL (*n* = 23)	+ 0.5 mL 2.25% epinephrine	2.5 mL (*n* = 23)	+ 0.5 mL 2.25% epinephrine	RDAI ROH ROR
Hou et al. 2016 [34] China	1 ~ 11 mon Inpatients *n* = 34	6 M (50.4%) No information	(*n* = 17) *how many milliliters not reported	+ 1.25 ml atrovent + 1 ml budesonide	(*n* = 17) *how many milliliters not reported	+ 1.25 ml atrovent + 1 ml budesonide	LOS TOS FOWITN
Ipek et al.2011 [35] Turkey	< 24 mon ED *n* = 120	7.9 mon (59.2%) No information	1) 4 mL (*n* = 30) 2) 4 mL (*n* = 30)	+ 0.15 mg/kg salbutamol	1) 4 mL (*n* = 30) 2) 4 mL (*n* = 30)	+ 0.15 mg/kg salbutamol	ROH
Islam et al. 2018 [36] Bangladesh	1 ~ 24 mon Inpatients *n* = 90	5.4 mon (56.6%) No information	4 mL (*n* = 45)		4 mL (*n* = 45)		LOS CSS
Kanjanapradap et al. 2018 [37] Thailand	6 mon~ 5 years Inpatients *n* = 47	20.1 mon (60%) 25.5%	3.5 mL (*n* = 22)	+ 2.5 mg salbutamol	3.5 mL (*n* = 25)	+ 2.5 mg salbutamol	
Khanal et al. 2015 [38] Nepal	6 wk. ~ 24 mon ED/OPD *n* = 100	9.7 M (48%) No information	4 mL (*n* = 50)	+ 1.5 mg epinephrine	4 mL (*n* = 50)	+ 1.5 mg epinephrine	ROR
Kose et al. 2016 [39] Turkey	1 ~ 24 mon Inpatients *n* = 70	7.6 mon (40.3%) No information	2.5 mL (*n* = 35)	+ 0.15 mg/kg salbutamol	2.5 mL (*n =* 35)	+ 0.15 mg/kg salbutamol	LOS CSS
Kuzik et al. 2007 [40] Canada	< 18 mon Inpatients *n* = 91	4.7 mon (59.4%) 68.5%	4 mL (*n* = 45)		4 mL (*n* = 46)		LOS
Kuzik et al. 2010 [41] Canada	< 24 mon ED *n* = 88	8.9 mon (77.5%) 47% History of asthma	4 mL (*n* = 44)	+ 1 mg salbutamol	4 mL (*n* = 44)	+ 1 mg salbutamol	RDAI ROH
Li et al. 2014 [42] China	2 ~ 18 mon OPD *n* = 84	7.2 mon (73.3%) No information	2 mL (*n* = 42)		2 mL (*n* = 42)		CSS
Luo et al. 2010 [44] China	< 24 mon Inpatients *n* = 93	5.8 mon (60.2%) 69.9%	4 mL (*n* = 50)	+ 2.5 mg salbutamol	4 mL (*n* = 43)	+ 2.5 mg salbutamol	LOS CSS
Luo et al. 2011 [43] China	< 24 mon Inpatients *n* = 112	5.9 mon (56.3%) 73.2%	4 mL (*n* = 57)		4 mL (*n* = 55)		LOS CSS
Mahesh Kumar et al. 2013 [45] India	< 24 mon Inpatients *n* = 40	5.9 mon (62.5%) No information	3 mL (*n* = 20)	+ 0.15 mg/kg albuterol	3 mL (*n* = 20)	+ 0.15 mg/kg albuterol	LOS
Mandelberg et al. 2003 [46] Israel	< 12 mon Inpatients *n* = 52	2.9 mon (57.7%) 86.5%	4 mL (*n* = 27)	+ 1.5 mg epinephrine	4 mL (*n* = 25)	+ 1.5 mg epinephrine	LOS CSS
Miraglia et al. 2012 [47] Italy	< 24 mon Inpatients *n* = 106	4.5 mon (65.1%) 82%	4 mL (*n* = 52)	+ 1.5 mg epinephrine	4 mL (*n* = 54)	+ 1.5 mg epinephrine	LOS CSS
Morikawa et al. 2018 [48] Japan	< 12 mon Inpatients *n* = 128	4.3 mon (39.2%) No information	2 mL (*n* = 63)	+ 0.5% 0.1 mL salbutamol	2 mL (*n* = 65)	+ 0.5% 0.1 mL salbutamol	LOS
Ojha et al. 2014 [49] Nepal	6 wk. ~ 24 mon Inpatients *n* = 59	8.5 mon (74%) No information	4 mL (*n* = 28)		4 mL (*n* = 31)		LOS
Pandit et al. 2013 [50] India	2 ~ 12 mon Inpatients *n* = 100	Not reported (Not reported) No information	4 mL (*n* = 51)	+ 1 mL adrenaline	4 mL (*n* = 49)	+ 1 mL adrenaline	LOS
Ratajczyk-Pekrul et al. 2016 [51] Poland	< 18 mon Inpatients *n* = 78	4.9 mon (58.9%) 53.5%	3 mL (*n* = 41)	+ 0.15 mg/kg salbutamol	3 mL (*n* = 37)	+ 0.15 mg/kg salbutamol	LOS
Sarrell et al. 2002 [52] Israel	< 24 mon OPD *n* = 65	12.5 mon (59%) 80%	2 mL (*n* = 33)	+ 5 mg terbutaline	2 mL (*n* = 32)	+ 5 mg terbutaline	ROH
Sharma et al. 2013 [53] India	1 ~ 24 mon Inpatients *n* = 248	8.5 mon (76.2%) No information	4 mL (*n* = 125)	+ 2.5 mg salbutamol	4 mL (*n* = 123)	+ 2.5 mg salbutamol	LOS
Silver et al. 2015 [54] USA	< 12 mon Inpatients *n* = 190	4.2 mon (61%) 67.5% History of asthma	4 mL (*n* = 93)		4 mL (*n* = 97)		ROR
Tal et al. 2006 [55] Israel	< 24 mon Inpatients *n* = 41	2.6 mon (56.1%) 80.5%	4 mL (*n* = 21)	+ 1.5 mg epinephrine	4 mL (*n* = 20)	+ 1.5 mg epinephrine	LOS CSS
Teunissen et al. 2014 [56] The Netherlands	< 24 mon Inpatients *n* = 164	3.4 mon (57.1%) 86.2%	4 mL (*n* = 84)	+ 2.5 mg salbutamol	4 mL (*n* = 80)	+ 2.5 mg salbutamol	LOS
Wang et al. 2014 [57] China	2 ~ 14 mon Inpatients *n* = 76	5.8 mon (56.6%) No information	2 mL (*n* = 37)	+ 0.5 ml salbutamol + 0.5 mg budesonide	2 mL (*n* = 39)	+ 0.5 ml salbutamol + 0.5 mg budesonide	LOS TOS FOWITN
Wu et al. 2014 [58] USA	< 24 mon ED *n* = 408	6.5 mon (56.8%) 62.4%	4 mL (*n* = 211)		4 mL (*n* = 197)		RDAI ROH

1. ED, emergency department; OPD, outpatient department; RSV, respiratory syncytial virus

2. 3% HS, 3% hypertonic saline; 0.9% NS, 0.9% normal saline

3. Epinephrine: Adrenaline; Atrovent, Salbutamol, Albuterol, Terbutaline: Bronchodilator; Budesonide: Corticosteroids

4. CSS, clinical severity score; RDAI, respiratory distress assessment instrument; LOS, length of hospital stay; ROH, rate of hospitalization; ROR, rate of re-admission; TOS, time of sleeping; FOWITN, frequency of waking up in the night

### Quality assessment of the included literature

**Fig. 2:**
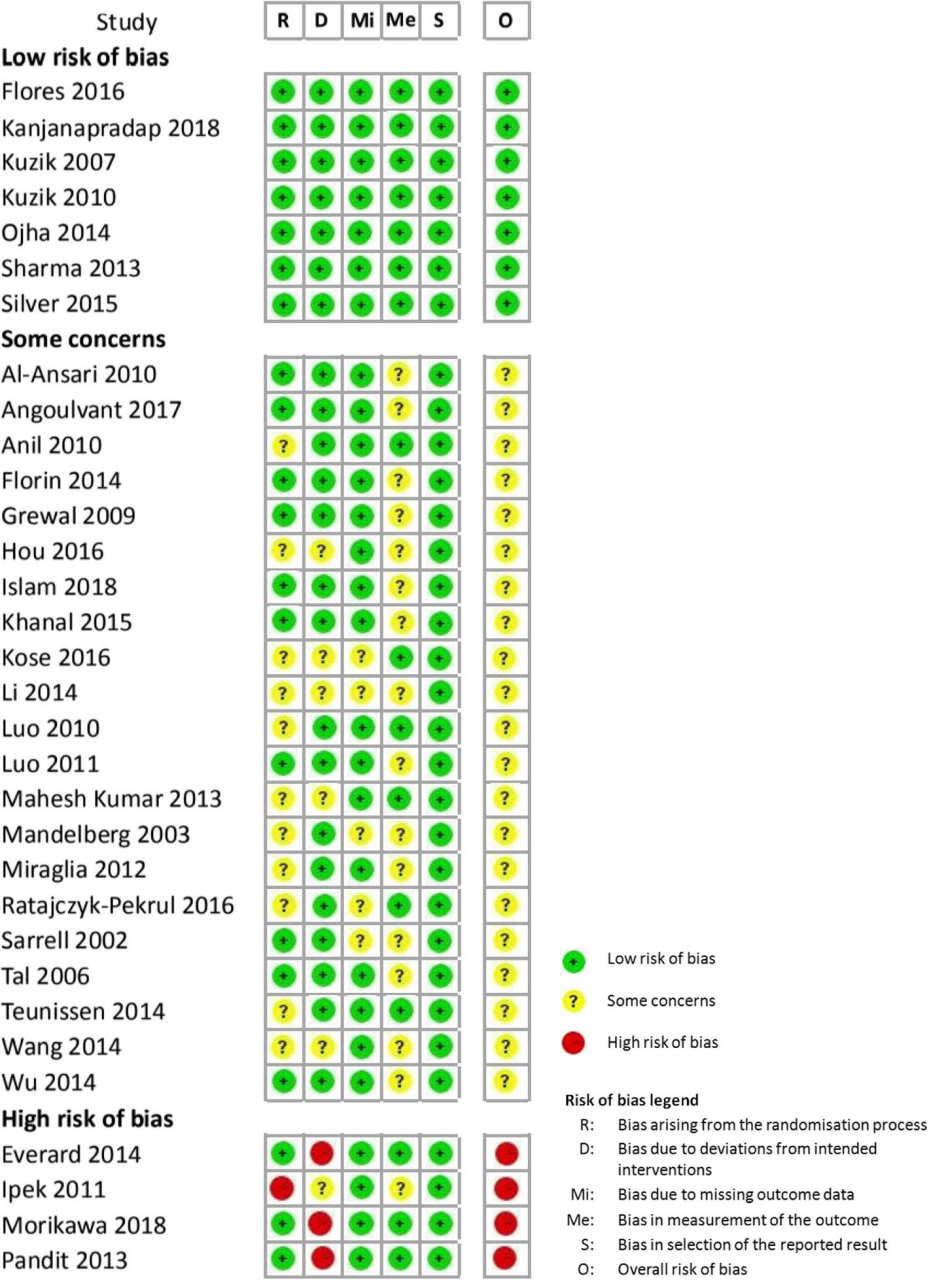
Risk of bias assessment for the included studies

According to the Cochrane risk of bias tool 2.0, quality assessment results of the included literature showed the following results: (1) For bias arising from the randomization process, 20 (62.5%) studies used the computer for random grouping and used a light-proof envelope to keep the groups hidden during the process; 11 studies (34.4%) did not clearly explain randomization or hidden process, whereas one study (3.1%) grouped subjects according to the order of admission, which did not meet randomization requirements and was assessed to be with high risk of bias. (2) For bias due to deviations from the intended intervention, both subjects and caretakers in 23 studies (71.9%) were blinded, six studies (18.8%) had no information on whether blinding was performed, and three studies (9.4%) indicated that neither subjects nor caretakers were blinded, and therefore, these were assessed to be with high risk of bias. (3) For bias due to missing outcome data, 20 studies (62.5%) conformed to the intention-to-treat principle, and although there were certain data losses during the study process, those did not affect the balance of the subjects’ basic characteristics, and these were determined to be with low risk of bias; five studies (15.6%) had no information on whether loss of data affected the results, and these were assessed to be with some concern of bias. (4) For bias in measurement of the outcome, research personnel were the ones who measured the severity of respiratory distress, and it was not explained whether the evaluators were blinded. Thus, this could have caused some bias in measurement outcomes, and it was assessed to be with some concern of bias. (5) No situations of bias in selection of the reported results were found in the included articles, and the articles were assessed to be with low risk of bias. Finally, for overall assessment, seven studies (21.9%) showed low risk of bias, 21 studies (65.6%) showed some concern of bias, and four studies (12.5%) showed high risk of bias. Overall assessment result of the literature was some concern of bias, the details of which are demonstrated in Fig. 2.

GRADE was used to assess the evidence body of the included literature. The study included RCTs such that the starting evidence grade was high. However, regarding the severity level of respiratory distress, the evidence level was degraded considering that the overall risk assessment results indicated some concern about bias. With regards to the severity of respiratory distress, the Clinical Severity Score (CSS) was used to assess the severity of respiratory distress, and a forest plot was used to demonstrate the high heterogeneity (*I*^2^ > 75%). Thus, the evidence level was degraded owing to inconsistency. Regarding the LOS, considering that the overall risk assessment results showed bias with some concern and the forest plot also showed high heterogeneity (*I*^2^ > 75%), the evidence level was degraded owing to risk of bias and inconsistency, and the overall evidence level was moderately low, with details summarized in Table 2. Lastly, in accordance with the evidence that the intervention measure could significantly improve the severity of respiratory distress and shorten the LOS while causing no severe adverse effects, results showed that the 3% HS benefits outweighed the risks, and this practice could be strongly recommended.

**Table 2:** Summary of findings using GRADE

**Summary of findings:**						
**3% Hypertonic Saline compared to 0.9% Normal Saline for ped bronchitis**						
**Patient or population**: ped bronchitis **Setting**: **Intervention**: 3% Hypertonic Saline **Comparison**: 0.9% Normal Saline						
Outcomes	Anticipated absolute effects^*^ (95% CI)		Relative effect(95% CI)	№ of participants (studies)	Certainty of the evidence(GRADE)	Comments
	**Risk with 0.9% Normal Saline**	**Risk with 3% Hypertonic Saline**				
CSS	The mean CSS was −3.57 to 8.8 point	MD 0.93 point lower(1.23 lower to 0.62 lower)	–	2010(11 RCTs)	⨁⨁◯◯LOW ^a,b^	
RDAI	The mean RDAI was −4.7 to 5.32 point	MD 0.6 point lower(0.95 lower to 0.26 lower)	–	1369(5 RCTs)	⨁⨁⨁◯MODERATE ^a^	
LOS	The mean LOS was 1.4 to 7.49 days	MD 0.54 days lower(0.86 lower to 0.23 lower)	–	2055(20 RCTs)	⨁⨁◯◯LOW ^a,b^	
Rate of hospitalisation	402 per 1000	342 per 1000(298 to 394)	**RR 0.85**(0.74 to 0.98)	1710(8 RCTs)	⨁⨁⨁◯MODERATE ^a^	
Rate of re-admission	135 per 1000	97 per 1000(53 to 180)	**RR 0.72**(0.39 to 1.33)	485(4 RCTs)	⨁⨁⨁◯MODERATE ^a^	
Time of sleeping	The mean time of sleeping was 4.54 to 7.32 h	MD 1.72 h higher(0.43 lower to 3.88 higher)	–	110(2 RCTs)	⨁⨁◯◯LOW ^a,b^	
Frequency of wake-up in the night	The mean frequency of wake-up in the night was 3.11 to 9.28 time	MD 5.61 time lower(6.54 lower to 4.67 lower)	–	110(2 RCTs)	⨁⨁⨁◯MODERATE ^a^	

*The risk in the intervention group (and its 95% confidence interval) is based on the assumed risk in the comparison group and the relative effect of the intervention (and its 95% CI)

CI: Confidence interval; MD: Mean difference; RR: Risk ratio

GRADE Working Group grades of evidence

High certainty: We are very confident that the true effect lies close to that of the estimate of the effect

Moderate certainty: We are moderately confident in the effect estimate: The true effect is likely to be close to the estimate of the effect, but there is a possibility that it is substantially different

Low certainty: Our confidence in the effect estimate is limited: The true effect may be substantially different from the estimate of the effect

Very low certainty: We have very little confidence in the effect estimate: The true effect is likely to be substantially different from the estimate of effect

Explanations

a. The overall of Risk of Bias was some concern

b. I^2^ > 75% (statistically significant)

### Meta-analytical results

#### Primary results: severity of respiratory distress

Regarding the severity of respiratory distress, the included studies used the CSS and Respiratory Distress Assessment Instrument (RDAI) for evaluation.

#### Clinical severity score (CSS)

**Fig. 3:**
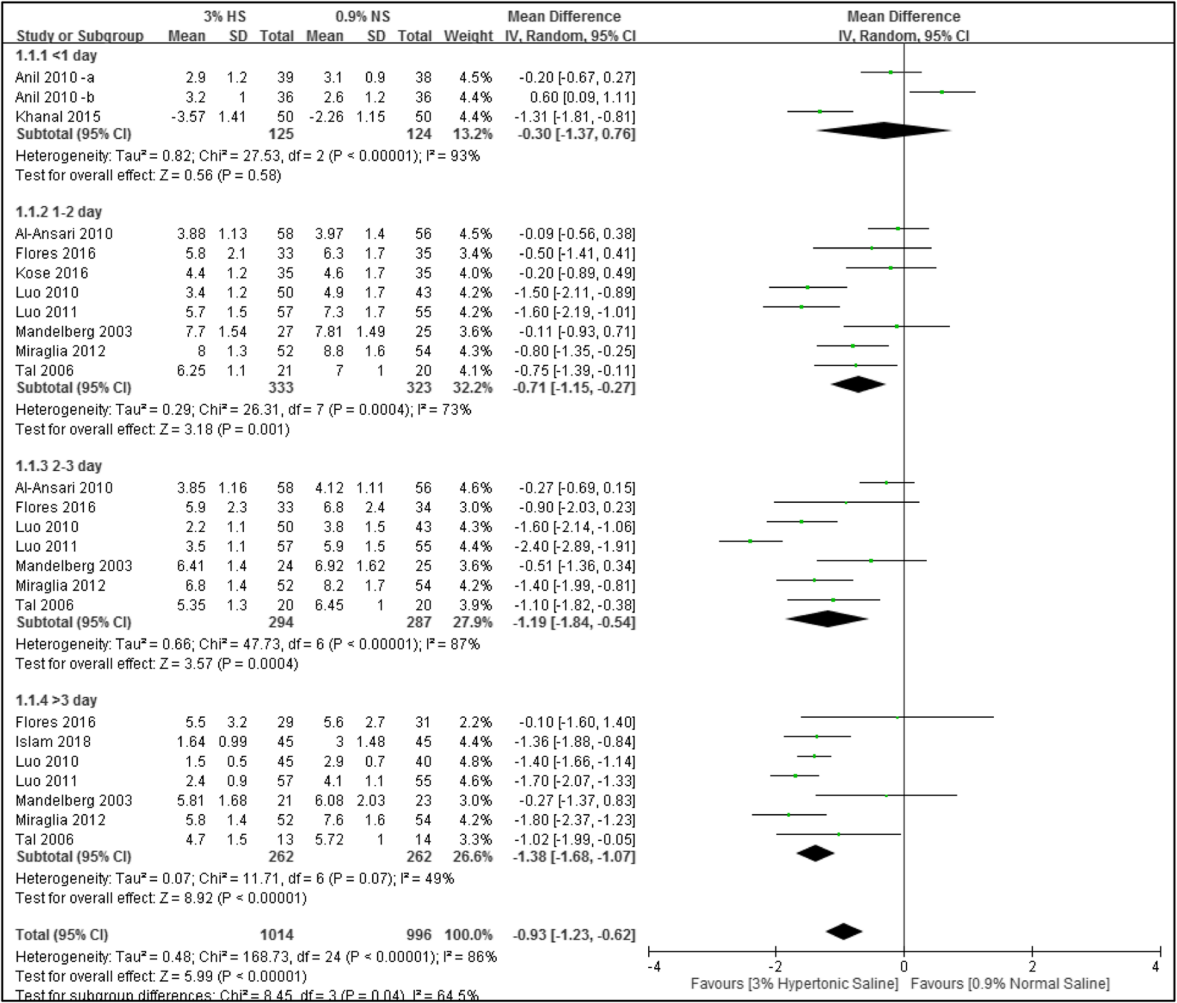
Forest plot of the clinical severity score (CSS)

In total, 11 studies used the CSS for evaluation. According to differences in days of measurement for each study (ranging 1 ~ 3 days), four subgroups were used for analysis as follow: < 1 day of measurement (*n* = 2, participants = 249), 1 or 2 days of measurement (*n* = 8, participants = 656), 2 or 3 days of measurement (*n* = 8, participants = 581), and > 3 days of measurement (*n* = 7, participants = 524). Results showed that compared to the group that used normal saline, the group that used 3% hypertonic saline for nebulizing treatment had significantly greater differences in the score for respiratory distress severity for the subgroups of 1 ~ 2, 2 ~ 3, and > 3 days with 0.71 points (*n* = 8; MD, − 0.71; 95% CI, − 1.15 to − 0.27; *I*^2^ = 73%), 1.19 points (n = 8; MD, − 1.19; 95% CI, − 1.84 to − 0.54; *I*^2^ = 87%), and 1.38 points (*n* = 7; MD, − 1.38; 95% CI, − 1.68 to − 1.07; *I*^2^ = 49%), respectively. Only the subgroup which had < 1 day of measurement did not show a statistically significant difference between the two test groups (MD, − 0.30; 95% CI, − 1.37 to 0.76, *I*^2^ = 93%). Data are shown in Fig. 3.

#### Respiratory distress assessment instrument (RDAI)

**Fig. 4:**
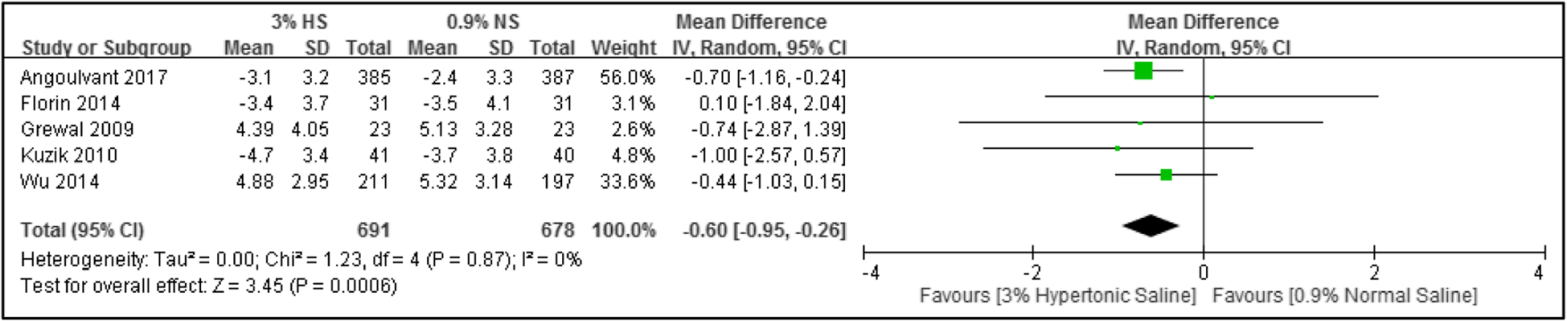
Forest plot of the Respiratory Distress Assessment Instrument (RDAI)

In total, five papers used the RDAI for evaluation. There were 1369 subjects in total, and the meta-analytical results showed that compared to the group that used normal saline, those used hypertonic saline for nebulizing treatment had a mean 0.6 points lower score of respiratory distress severity (*n* = 5; MD, − 0.60; 95% CI, − 0.95 to − 0.26; *I*^2^ = 0%), as demonstrated in Fig. 4.

### Secondary results

#### Length of hospital stay (LOS)

**Fig. 5:**
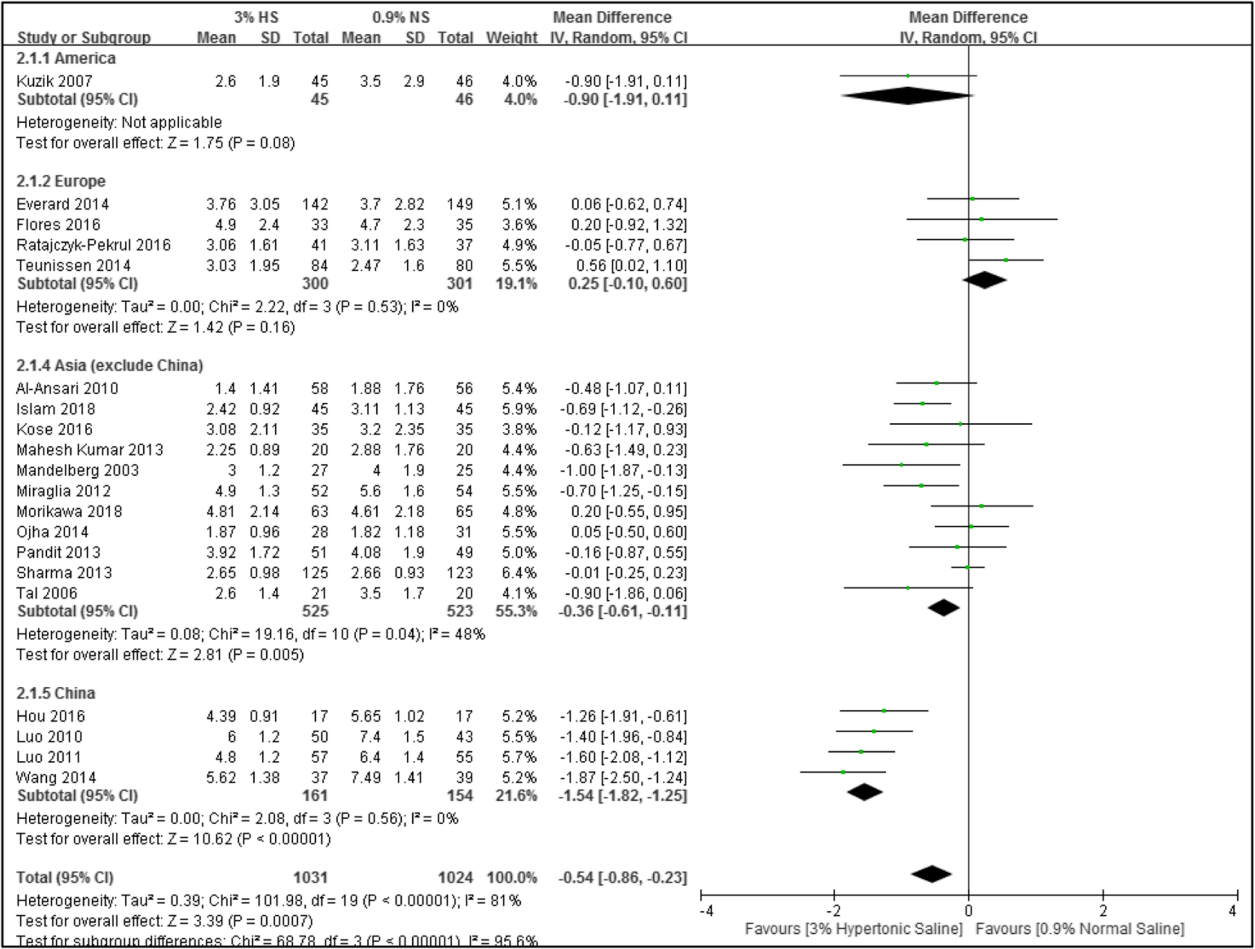
Forest plot of length of hospital stay (LOS)

**Fig. 6:**
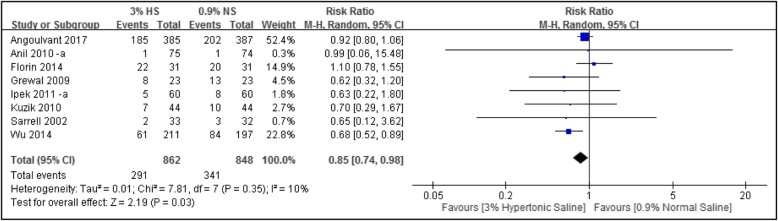
Forest plot of the rate of hospitalization

In total, 20 studies were included with 2055 subjects. Meta-analytical results showed that compared to the group using normal saline, the group using hypertonic saline for nebulizing treatment had a 0.54-day shorter LOS (*n* = 20; MD, − 0.54; 95% CI, − 0.86 to − 0.23; *I*^2^ = 81%), as demonstrated in Fig. 6. Because this result was highly heterogeneous, further subgroup analyses were performed with respect to different regions, which greatly reduced the heterogeneity: the Americas and Europe (*I*^2^ = 0%), Asia (excluding China) (*I*^2^ = 48%), and China (*I*^2^ = 0%), as demonstrated in Fig. 5.

#### Rate of hospitalization

In total, eight studies were included with 1710 subjects. Meta-analytical results showed that compared to the group using normal saline, the group using hypertonic saline for nebulizing treatment had a significant lower rate of hospitalization (*n* = 8; RR, 0.85; 95% CI, 0.74 to 0.98; *I*^2^ = 10%), as shown in Fig. 6.

#### Rate of readmission

**Fig. 7:**
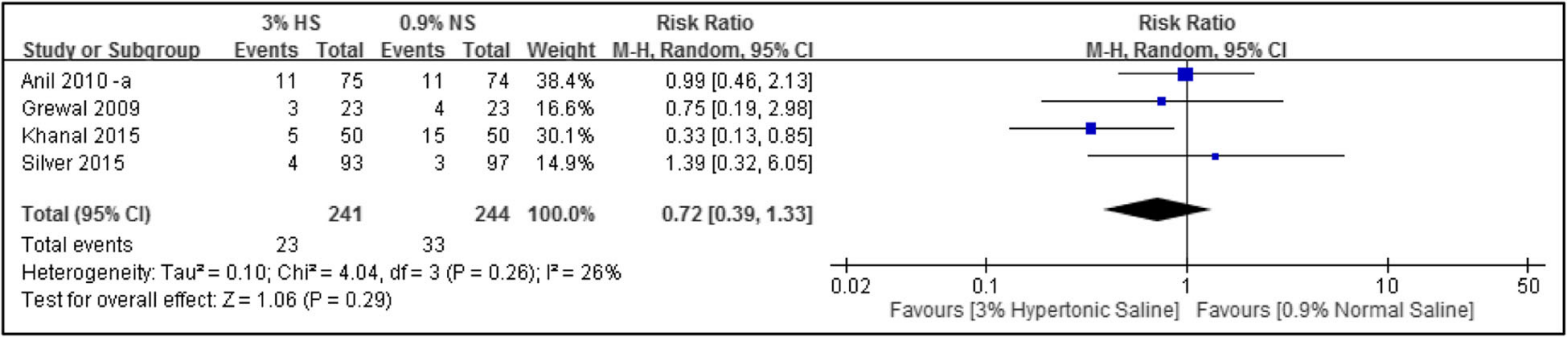
Forest plot of the rate of readmission

In total, four studies were included with 485 subjects. Meta-analytical results showed that compared to the group using normal saline, the group using hypertonic saline for nebulizing treatment had lower rates of readmission (*n* = 4; RR, 0.72; 95% CI, 0.39 to 1.33; *I*^2^ = 26%), but it did not reach statistical significance, as shown in Fig. 7.

#### Time of sleeping

**Fig. 8:**
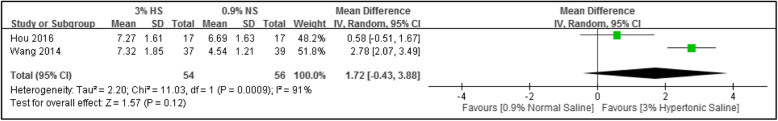
Forest plot of time of sleeping

Two studies were included with 110 subjects. Meta-analytical results showed that compared to the group using normal saline, the using hypertonic saline for nebulizing treatment had 1.72 h longer sleep time at night (*n* = 2; MD, 1.72; 95% CI, − 0.43 to 3.88; *I*^2^ = 91%), but this did not reach statistical significance, as shown in Fig. 8.

#### Frequency of waking up in the night

**Fig. 9:**
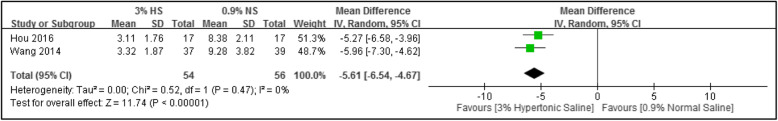
Forest plot of the frequency of waking up in the night

Two studies were included with 110 subjects. Meta-analytical results showed that compared to the group using normal saline, the using hypertonic saline for nebulizing treatment demonstrated effectively reduced the frequency of waking up in the night by five times (n = 2; MD, − 5.61; 95% CI, − 6.54 to − 4.67; *I*^2^ = 0%), as shown in Fig. 9.

#### Adverse events

Twelve studies reported mild adverse events, including cough [27, 31, 39, 54, 56, 58], bronchospasm [39, 56], vomiting and diarrhea [33, 50], desaturation [56], agitation [40, 56], rhinorrhea [27], tachycardia [57], hoarse voices [43], vigorous crying [40], vomiting and diarrhea [33, 50]. One study [26] reported adverse event (bradycardia and desaturation) in hypertonic saline group. However, these were mild and resolved naturally and all subjects completed the trial process.

### Sensitivity analysis results

Because the forest plot for LOS showed high heterogeneity, we conducted a sensitivity analysis regarding this and used research method differences (PICO) for a subgroup analysis based on whether there was combined use of other drugs. The results after grouping showed no significant effects on the overall results. However, when a subgroup analysis was done for different regions (Americas, Europe, China, and other Asian countries), it was found that the heterogeneity greatly decreased, and high heterogeneity existed among groups (*I*^2^ = 95.6%), demonstrating that this may be the cause for the heterogeneity.

### Analysis of publication

Because there were more than 10 trials in our systemic review, therefore we created and examined a funnel plot to explore possible publication bias. There appeared to be no evidence of publication bias in the included studies.

## Discussion

Results of the meta-analyses in this study showed that compared to the use of normal saline and regardless of whether or not children were hospitalized, the use of hypertonic saline for nebulizing treatment improved the severity of respiratory distress, extended the sleep time, reduced the frequency of waking up during the night, and shortened the children’s LOS. For non-hospitalized children, it also reduced the rate of hospitalization.

All subjects included in the trials were diagnosed with acute bronchiolitis, and there were no significant differences in the sex ratio. However, the severity of respiratory syncytial virus (RSV) infection was inconsistent, and this might have affected the effects of the interventions. Additionally, all subjects in the study were children aged < 2 years, and only one study included subjects aged between 6 months and 5 years old. However, the measurement results for respiratory distress severity in that particular study were recorded as median and quartiles and could not be included in data calculations. Therefore, that study was excluded from the meta-analysis. The study only included subjects aged < 2 years for analysis; therefore, additional research will be required to verify whether the study results are suitable for children aged > 2 years.

There were differences in the intervention measures in each of the studies included. The nebulization treatment time lasted for 20 ~ 30 min, but the saline dosage used for nebulizing ranged from 2 to 5 ml. In addition, for subjects with different clinical symptoms, most studies combined treatment with epinephrine, bronchodilators, or steroids. Although this may have affected the treatment results, it was an unavoidable variable owing to treatment needs. Regarding this, the study conducted subgroup analyses on the aforementioned two variables (saline dosage and drug combinations). It was found that neither of these variables were the cause of the high heterogeneity. Related literature also pointed out that combined drugs were not the primary reasons interfering with the efficacy of results [59–61].

The primary result in this study was respiratory distress severity. Results demonstrated no significant difference in disease improvement for < 1 day of nebulizing treatment; however, with a longer duration of nebulizing treatment with hypertonic saline, improvements in respiratory distress severity scores were more significant. We speculated the following two reasons could be the causes for this effect. First, it takes more than 1 day for hypertonic saline to reach its efficacy, after children are hospitalized for treatment, their autoimmunity and body strength recover along with an increase in the treatment duration. Second, the disease severity is gradually ameliorated along with the disease course, thus showing more-significant treatment efficacy [62–64].

The study results showed that those who used hypertonic saline for nebulizing treatment had 0.54 less day of LOS compared to those who used normal saline. It was statistically significant, although the amount of decline is small, and this is a huge breakthrough in hospitals where inpatients are saturated. Longer LOS was observed in the Chinese studies than studies conducted in other countries, might because of different local customs and insurance systems. For example, the hospitalization costs can be fully covered by health insurance among children who diagnosed with bronchiolitis. Therefore, caregivers may decide to discharge from hospital until children completely recovered. National cultural differences may be another factor [29, 42–44], but this would require further research for verification.

Sleep quality is relatively important for children’s mental and physical development [13–15]. This is the first study to analyze sleep quality (including sleep time and frequency of waking up at night) in children with bronchiolitis undergoing nebulizing treatment. Among the five studies of Chinese subjects included, only two investigated night-time sleep quality [34, 57]. In these two articles, it was stated that the sleep time and frequency of waking up at night (opening eyes as the calculation standard) were recorded by the nurse and family member from 8 pm to the next day 8 am. Results showed that hypertonic saline was effective in reducing the frequency of waking up at night. Although the results did not reach significance, it was a major breakthrough regarding investigation of sleep quality. We suggest that in the future clinical trials, it should include sleep quality as an index of measurements.

### Limitations

The study had three main limitations: (1) inconsistent disease severity in the included subjects; (2) differences between studies with respect to dosage of hypertonic saline used for the intervention and the combined use of drugs such as bronchodilators; and (3) evaluators of the severity of respiratory distress were either medical personnel or research personnel who were not blinded. All these factors may have affected the quality of the study results.

## Conclusions

Using hypertonic saline for nebulizing treatment in children with bronchiolitis can significantly improve the severity of respiratory distress, shorten the LOS, and increase the children’s night-time sleep quality. It is recommended that a large-scale randomized clinical trial with a standardized design be conducted in the future to investigate the effects of hypertonic saline in children with bronchiolitis.

## Data Availability

Not applicable.

## Abbreviations

ED: emergency department
OPD: outpatient department
RSV: respiratory syncytial virus
3% HS: 3% hypertonic saline
0.9% NS: 0.9% normal saline
CSS: clinical severity score
RDAI: respiratory distress assessment instrument
LOS: length of hospital stay
ROH: rate of hospitalization
ROR: rate of re-admission
TOS: time of sleeping
FOWITN: frequency of waking up in the night
